# Changes in T2 Relaxation Time Mapping of Intervertebral Discs Adjacent to Vertebrae after Kyphoplasty Correlate with the Physical Clinical Outcome of Patients

**DOI:** 10.3390/diagnostics12030605

**Published:** 2022-02-27

**Authors:** Lisa C. Wegener, Felix Werner, Arnd Kleyer, David Simon, Michael Uder, Rolf Janka, Siegfried Trattnig, Goetz H. Welsch, Milena L. Pachowsky

**Affiliations:** 1 Friedrich-Alexander-University Erlangen-Nürnberg (FAU), 91054 Erlangen, Germany; lisa_christin_wegener@web.de; 2 Johanna-Etienne Hospital, 41462 Neuss, Germany; 3 Department of Internal Medicine 4–Nephrology, University Hospital of Erlangen, Friedrich-Alexander-University Erlangen-Nürnberg (FAU), 91054 Erlangen, Germany; felix.werner@uk-erlangen.de; 4 Department of Internal Medicine 3–Rheumatology and Immunology, University Hospital of Erlangen, Friedrich-Alexander-University Erlangen-Nürnberg (FAU), 91054 Erlangen, Germany; arnd.kleyer@uk-erlangen.de (A.K.); david.simon@uk-erlangen.de (D.S.); 5 Institute of Radiology, University Hospital of Erlangen, Friedrich-Alexander-University Erlangen-Nürnberg (FAU), 91054 Erlangen, Germany; michael.uder@uk-erlangen.de (M.U.); rolf.janka@uk-erlangen.de (R.J.); 6 High Field MR Center, Department of Biomedical Imaging and Image Guided Therapy, Medical University of Vienna, 1090 Vienna, Austria; siegfried.trattnig@akhwien.at; 7 UKE Athleticum, Department of Trauma and Orthopedic Surgery, University Hospital Hamburg-Eppendorf, University Medical Centre Hamburg-Eppendorf, 20251 Hamburg, Germany; g.welsch@uke.de; 8 Department of Internal Medicine 3–Rheumatology and Immunology, Department of Trauma and Orthopaedic Surgery, University Hospital of Erlangen, Friedrich-Alexander-University Erlangen-Nürnberg (FAU), 91054 Erlangen, Germany

**Keywords:** intervertebral disc, MRI, T2 mapping, kyphoplasty, clinical outcome

## Abstract

(1) Background: To assess whether clinical outcomes correlate with tissue changes in the intervertebral discs (IVDs) after kyphoplasty as treatment for vertebral fractures, quantitative MRI was applied. (2) Methods: Quantitative T2 mapping acquired in a 3 T MRI scanner of the thoracolumbar spine was performed in 20 patients two years after kyphoplasty. The IVDs adjacent and nonadjacent to the treated vertebrae were divided into six regions of interest (ROI), which were further categorised into inner (ROI 2–5) and outer (ROI 1 and 6) parts of the IVDs, and the T2 values were analysed. T2 values of adjacent discs were correlated with the items of questionnaires evaluating the clinical outcome (i.e., 36-Item Short Form Survey). (3) Results: Lower T2 values in adjacent IVDs correlated with poorer physical outcome two years after kyphoplasty. The inner part of the IVDs adjacent to treated vertebrae showed statistically significant lower T2 values in segments L2/L3 and L3/L4 compared to nonadjacent ones. Patients with lower T2 values showed more pain and physical limitations in everyday life. (4) Conclusions: Quantitative T2 mapping can detect IVD degeneration in patients after kyphoplasty and correlates with the physical outcome. This technique could help to gain better insights into alterations in tissue composition following kyphoplasty and the consequences for the patients’ quality of life.

## 1. Introduction

Due to demographic changes, in the following decades, there will be a growing number of older people [1,2]. Therefore, a higher quantity of vertebral compression fractures resulting from osteoporosis or non-osteoporotic accidental fractures can be expected [3,4,5,6,7]. This will potentially lead to decreased quality of life for patients and to increased costs for the medical health care systems [1,7]. Thus, it is important to accommodate patients with the best supportive care. The choice of adequate therapy is crucial. Besides non-surgical therapy and prevention of fractures, surgical interventions are gaining importance. Vertebroplasty and kyphoplasty are surgical techniques used to treat vertebral compression fractures (VCF) with little or no posterior wall involvement. Both techniques are minimally invasive and “involve injection of cement (polymethylmethacrylate [PMMA] into the fractured vertebral body” [8]. For kyphoplasty, there is an “additional step of cavity creation; most typically, a balloon tamp is inflated to create a cavity into which PMMA is injected” [8]. These surgical techniques are implemented in patients with pain and disability [8,9]. Both procedures show only subtle differences in the clinical outcome in long-term follow-up studies and are regarded as safe and effective [9,10]. On top of that, kyphoplasty is considered superior to vertebroplasty regarding “the injected cement volume, the short-term pain relief, the improvement of short- and long-term kyphotic angle, and lower cement leakage rate” [9]. Zitka et al. [11] stated in their study that kyphoplasty is a suitable surgical technique to reduce pain and maintain functional status in older patients. However, there are also very critical views of kyphoplasty; not all patients profit satisfactorily from this surgical intervention and there is little knowledge about the effect of kyphoplasty on the intervertebral discs (IVDs) [12]. 

To detect subtle changes in IVDs and vertebrae in the thoracolumbar spine, magnetic resonance imaging (MRI) is the most sensitive and non-invasive diagnostic device at present and can be regarded as the gold standard [13,14]. MRI allows better visualisation of internal organs compared to computed tomography (CT), which, unlike MRI, uses radioactive radiation [15]. MRI images are generated using a strong magnetic field and radio frequency waves [15]. Recently, T2 mapping has been gaining importance in the non-invasive quantitative evaluation of IVDs by characterising different degrees of intervertebral disc degeneration (IVDD) [16]. T2 mapping is based on the physical properties of relaxation of nuclei at the atomic level [17]. To create a T2 map from the different relaxation times, successive images with different echo times and signal levels are needed [17]. This technique is increasingly applied in detecting lesions in tissues and organs [18,19,20]. T2 mapping enables the analysis of cartilage hydration and collagen integrity without the need for contrast enhancement. T2 mapping has therefore proven to be a sensitive tool for the determination of cartilage damage in joints [21]. IVDs are also predestined for analysis with T2 mapping due to their structure of cartilaginous tissue. Water and collagen content and their changes in IVDs and other tissues can be investigated with T2 mapping [22].

A broader knowledge of postoperative changes after kyphoplasty will be helpful for a deeper understanding of structural and clinical outcomes after this surgical technique. In a previous study, the degeneration of adjacent and nonadjacent IVDs in the thoracic and lumbar spine approximately two years after kyphoplasty was examined by using quantitative MRI and changes in T2 relaxation time mapping. On average, degenerated parts showed reduced T2 values in IVDs adjacent to affected vertebrae compared to nonadjacent IVDs [23]. 

In this feasibility study, the aim was to analyse whether these changes in T2 relaxation times of the IVDs correlate with the clinical physical outcome in patients who had been treated with kyphoplasty. 

## 2. Materials and Methods

For this study, an ethical agreement was given by the Clinical Ethics Committee of Friedrich-Alexander-University in Erlangen, Germany. Written informed consent was obtained from all participating patients. All methods were performed in accordance with the relevant guidelines and regulations.

### 2.1. Patients and Procedure

There were 88 patients within the defined period of six months who had suffered a fracture of the spine between the segments of TH11 to S1 and had undergone kyphoplasty. The fractures treated by kyphoplasty were injuries of the vertebral body with little or no posterior wall involvement. For the categorisation of the different fracture types, the Classification of Osteoporotic Thoracolumbar Spine Fractures [24] was applied.

For each kyphoplasty, the respective vertebral body was accessed via a bilateral pedicle approach. For augmentation of the vertebral body, KYPHON^TM^ XPEDE bone cement was used. The volume of cement injected into the vertebral body was between 2 and 4 mL per side, i.e., between 4 and 8 mL per vertebral body were used.

**Figure 1:**
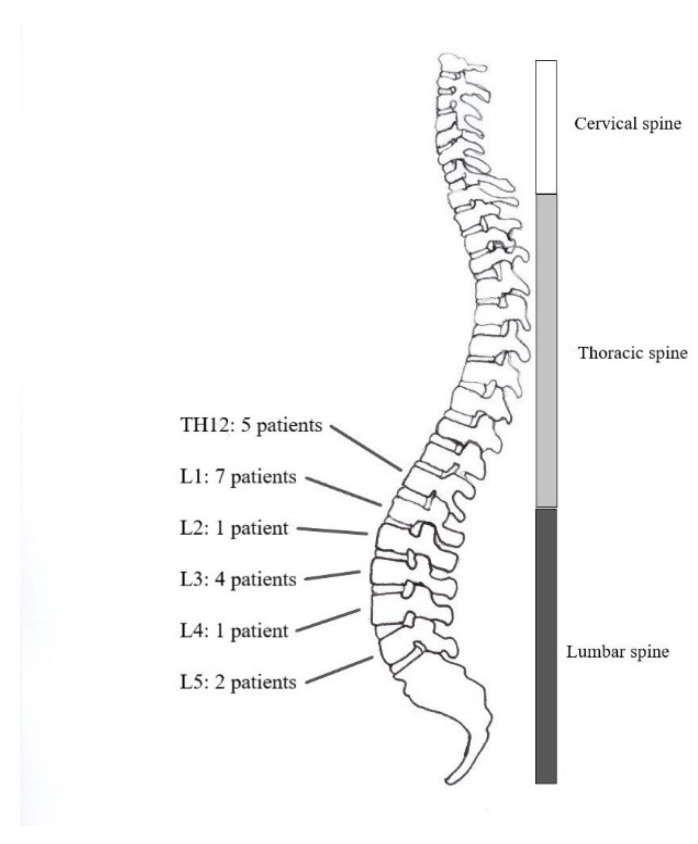
Number and distribution of fractured vertebral bodies per segment with kyphoplasty.

Clinical examination and diagnostic imaging indicated the necessity of surgery. In total, 68 patients had to be excluded from the follow-up study. Among the reasons for exclusion were the following: a high grade of disc herniation (15), immobility (6), severe comorbidity such as dementia (7), claustrophobia (8) and contraindications against MRI, i.e., metal implants or pacemaker (16). Ten potential patients did not consent to participate in the study. Six patients passed away before acquisition time. Out of the above-mentioned cohort, 20 patients agreed to join the study and were scheduled for MRI and clinical examination. The distribution of fractured vertebrae in the examined group of patients is illustrated in Figure 1.

### 2.2. Image Acquisition

For MRI image acquisition, a 3.0 Tesla (T) MR scanner (Magnetom Skyra, Siemens Medical Solution) with a gradient strength of 40 mT/m and a dedicated eight-channel spine coil (3 T Spine Matrix Coil, Siemens) was used. To acquire the MRI T2 images, a sagittal, multi-echo and “spine-echo” (SE) protocol was used. We utilised a multi echo-spine sequence for T2 relaxation time measurement (TR: 1400 ms, TE: 11.9, 23.8, 35.7, 47.6, 59.5, 71.4, 83.3, 95.2, 107.1, 119, 130.9, 142.8 ms, FOV: 260 × 260 mm, flip angle: 180°, pixel size: 0.4 × 0.4 mm^2^, slice thickness: 5 mm, receive bandwidth: 200 Hz/pixel, total acquisition time: 6:18 min). The T2 relaxation times were gained from inline reconstruction T2 maps by pixel-wise, mono-exponential, non-negative least squares (NNLS) fit analysis (MapIt, Siemens Medical Solution, Erlangen, Germany). On top of that, a T2 turbo-spin echo (TSE) sequence to acquire high-resolution morphological MRI (TR: 3500.0 ms, TE: 91.0 ms, FOV: 260 × 260 mm, flip angle: 160°, voxel size: 0.4 × 0.4 × 5.0 mm, acquisition time: 3:46 min) was applied. The sequences were all obtained in the sagittal plane.

### 2.3. Image Analysis

**Figure 2:**
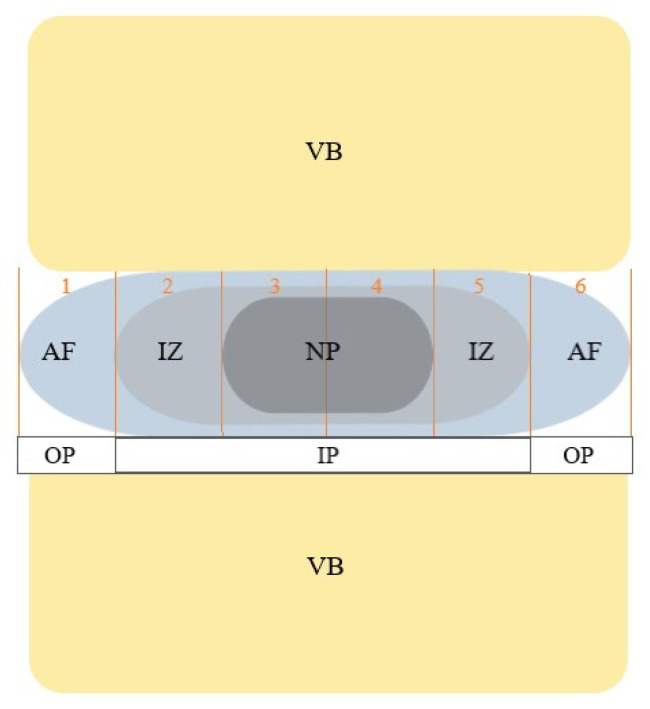
Scheme of the intervertebral disc (sagittal plane). The IVD consists of the nucleus pulposus (NP), the intermediate zone (IZ) and the anulus fibrosus (AF). The inner part (IP) contains the NP and IZ and the outer part (OP) contains the AF. The red lines and numbers 1 to 6 describe the different ROIs for T2 analysis of the IVD. The numbers 1 and 6 represent ROI 1 and ROI 6 and are classified as AF. The NP describes the inner part of the IVD as ROI 3 and ROI 4 and is marked in this figure as numbers 3 and 4. The IZ in between the AF and NP is shown by numbers 2 and 5 and displays ROI 2 and ROI 5.

**Figure 3:**
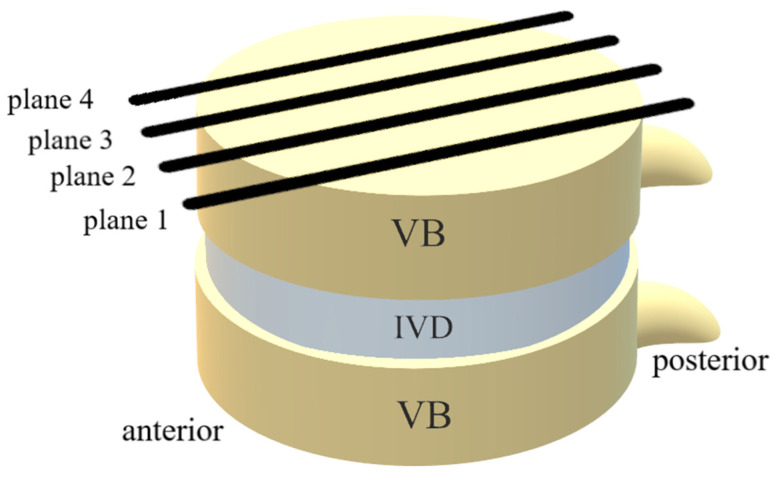
Scheme of the distribution of sagittal MR planes through the vertebral bodies (VB) and intervertebral discs (IVD). Planes 1 and 2 are located through the left part, planes 3 and 4 through the right part.

The MRI dataset was transmitted to a Syngo (Leonardo) workstation (Siemens Medical Solution, Erlangen, Germany) for analysis of the regions of interest (ROIs). By using a NNLS fit analysis, T2 relaxation time maps were obtained. At first, the MRI images were inspected for new fractures, leakage of cement into adjacent discs and motion artefacts, as well as disc bulging in the TSE images. The margins of vertebral bodies and the spinous process were used as anatomical landmarks. As there is no automatic analysis software available yet, each of the IVDs between TH11 and S1 were divided into 6 equal regions by our image analysts in each of the four sagittal planes (see Figure 2). Two planes were located through the left part of the IVDs and the other two through the right part (see Figure 3). Afterwards the ROIs, which were drawn on the TSE-sequenced MRI images before, were transferred onto the T2 maps. T2 relaxation times were collected for each ROI 1 to 6 of all IVDs between TH11 and S1 in the four planes, and mean values plus standard deviation were generated. The anterior and posterior ROIs (ROI 1 and ROI 6) were referred to as the anulus fibrosus (AF) and the inner ROIs (ROI 3 and ROI 4) as the nucleus pulposus (NP). The ROIs between AF and NP were labelled as the intermediate zone (IZ) (see Figure 2). 

To allow a comparison of the adjacent to nonadjacent IVDs of fractured vertebral bodies which had been treated with kyphoplasty, we created an internal control group within the spine. IVDs which were directly located above or beneath a fractured vertebra represented the adjacent IVDs and constituted group 1. Group 2 was established as an internal control group and consisted of all IVDs nonadjacent, i.e., with no direct contact, to a fractured vertebral body. For further analysis based on the clinical outcome established by means of the results of the questionnaires, NP and IZ were combined to an inner zone of the IVD. Thus, we created two ROI groups consisting of the outer part (OP) and the inner part (IP) of IVDs (see Figure 2).

### 2.4. Quality of Life Analysis

To collect data relating to the quality of life after kyphoplasty, several surveys, i.e., the 36-Item Short Form Survey (SF-36), the Oswestry Low Back Pain Questionnaire (ODQ) and the Numeric Rating Scale (NRS), were performed on the same day as the MRI acquisition.

#### 2.4.1. 36-Item Short Form Survey (SF-36)

The SF-36 is a widely used and well-validated survey for measuring the health-related quality of life [25,26]. It contains eight scales: vitality (VT), physical functioning (PF), bodily pain (BP), general health perceptions (GH), physical role functioning (RP), emotional role functioning (RE), social role functioning (SF) and mental health (MH) [27]. The Physical Component Summary (PCS) and the Mental Component Summary (MCS) are the summary scores of the different items of the questionnaire and allow a distribution into two dimensions [26].

#### 2.4.2. Oswestry Low Back Pain Disability Questionnaire (ODQ)

The ODQ measures the impact of low back pain on daily life and shows the dimension of disability “in people with acute, subacute, or chronic low back pain” [28]. There are 10 parameters enabling the calculation of the disability score: pain intensity, personal care, lifting, walking, sitting, standing, sleeping, sex life, social life and travelling [29]. “Each item is measured on a 6-point ordinal scale, ranging from the best scenario to the worst scenario” [28]. Percentages from 0% to 20% point to a minimal disability, 21% to 40% to a moderate disability, 41% to 60% to a severe disability, 61% to 80% to impairment in all areas of life and 81% to 100% to confinement to bed [28,29].

#### 2.4.3. Numerical Rating Scale (NRS)

The NRS is a one-dimensional metric rating scale for pain intensity and expresses the subjectively felt pain. The scale contains numbers from 0 to 10, with 0 representing no pain and 10 the most intense imaginable pain. The NRS is a valid and reliable scale widely used in clinical examination [30].

### 2.5. Statistical Analysis

For statistical analysis, SPSS software (IBM SPSS 25.0, IBM Corp., Armonk, NY, USA) was used. If not stated otherwise, continuous variables are provided as mean and standard deviation (SD).

The T2 relaxation times were compared between adjacent and nonadjacent IVDs for each ROI. Therefore, at first, quantile–quantile plots, Kolmogorov–Smirnov tests and Shapiro–Wilk tests were used to test the hypothesis of normally distributed continuous variables. Afterwards, the Gaussian distribution was applied. For independent samples, the Mann–Whitney U test or the t-test proved to be appropriate for further analysis.

Regression analysis was performed between the T2 values of the IP and OP of upper and lower adjacent IVDs and the subscales and summary scores of the SF-36 and the NRS. We correlated the items of the ODQ with T2 relaxation times of the IP and OP of upper and lower IVDs adjacent to fractured vertebrae. For further analysis, the study cohort was grouped according to gender (male, female), age (age < 70, age > 70) and body mass index (BMI) (BMI < 25, BMI > 25).

We considered *p*-values ≤ 0.05 as statistically significant.

## 3. Results

### 3.1. Patient Characteristics

Our study population consisted of 20 participants (14 females, 6 males). The mean age at surgery was 67.45 ± 8.49 years. An average of 24.20 ± 5.72 months had passed between surgery and the MRI scan. Body height was 1.70 ± 0.09 m on average, the mean weight was 73.45 ± 15.40 kg and the mean body mass index (BMI) was 25.63 ± 4.43 kg/m^2^.

All patients fully replied to the SF-36, the ODQ and the NRS before MRI examination.

### 3.2. T2 Mapping

**Figure 4:**
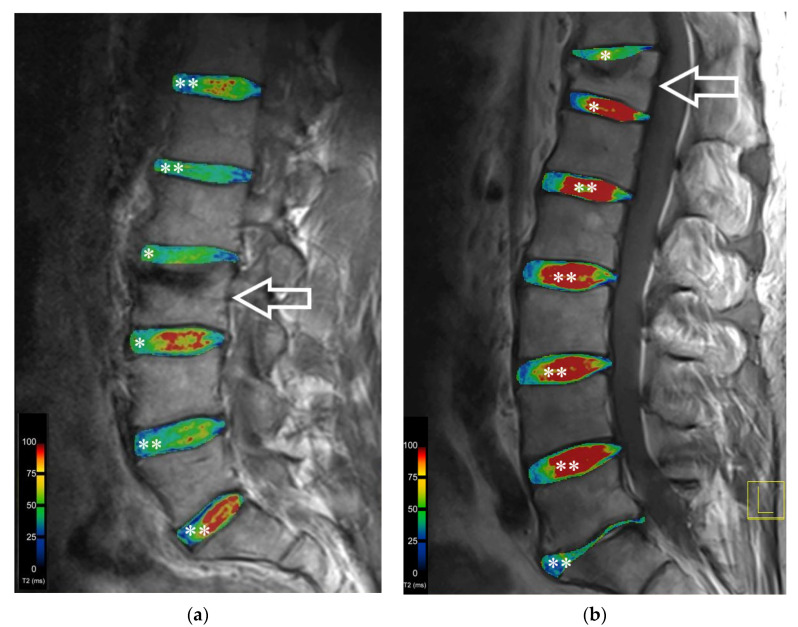
Sagittal TSE MR image and the corresponding colour-coded T2 map of the intervertebral disc. Sagittal TSE MR image and the corresponding colour-coded T2 map of the IVD of two different patients ((**a**) female, (**b**) male) show clearly visible differences between the upper intervertebral discs adjacent (*), especially upper IVD, and nonadjacent (**) to vertebral bodies after kyphoplasty. The arrow marks the vertebral body treated with kyphoplasty. The T2 values are represented by the colour-coded map within the IVDs, as shown in the colour bar.

T2 relaxation times in the adjacent IVDs were lower than in the nonadjacent IVDs in segments L2/L3 and L3/L4 in the NP and the IZ. These differences in segments L2/L3 and L3/L4 in the IP (ROI 2, ROI 3, ROI4 and ROI 5) showed statistically significantly lower values in T2 relaxation times in the adjacent IVDs compared to nonadjacent ones (see Table 1 and Figure 4). Segment L3/L4 also showed significantly lower values in the adjacent IVDs in ROI 3, ROI 4 and ROI 5 (see Table 1). In the OP, a statistically significant difference in T2 values between adjacent and nonadjacent IVDs was seen in segment TH12/L1 (see Table 2).

### 3.3. Quality of Life Analysis

#### 3.3.1. 36-Item Short Form Survey

For a complete overview of the values for all SF-36 items, see Table 3.

For patients older than 70 years, we found a statistically significant positive correlation between VT and the T2 values of upper IVDs adjacent to vertebrae treated with kyphoplasty. We also found a statistically significant positive correlation between VT and T2 values of upper adjacent IVDs in patients with a BMI < 25kg/m^2^.

In our study cohort, a statistically positive correlation between T2 values of upper adjacent IVDs and the score of PF was seen.

In patients with a BMI lower than 25 kg/m^2^ and in patients older than 70 years, we saw a statistically significant positive correlation between the item GH and T2 values of upper adjacent IVDs. 

The summary score PCS showed a statistically significant positive correlation with T2 values of upper IVDs adjacent to vertebrae treated with kyphoplasty in patients older than 70 years. 

#### 3.3.2. Oswestry Low Back Pain Disability Questionnaire and Disability Score

All values of this questionnaire are displayed in Table 4.

In this study cohort, in patients older than 70 years, a statistically significant negative correlation between “pain intensity” and T2 values of upper adjacent IVDs was found. 

Between the item “personal care” and T2 values of upper adjacent IVDs, a negative correlation could be shown in male patients. “Lifting” was negatively correlated with T2 values of upper IVDs adjacent to treated vertebrae in male patients older than 70 years. We found a negative correlation between the item “standing” and T2 values of upper adjacent IVDs. Statistically, a negative correlation between the item “sitting” and the T2 values of lower adjacent IVDs could be found.

#### 3.3.3. Numeric Rating Scale (NRS)

There was a positive correlation between the NRS and the age at surgery in female patients of our study population. A higher pain intensity according to the NRS was associated with lower T2 values of the OP in the upper adjacent IVDs in the male group, represented by a statistically negative correlation between these values. The mean value for NRS was 4.30 ± 2.03.

## 4. Discussion

The assessment of the clinical outcome of patients two years after kyphoplasty and the interrelation regarding changes in the ultrastructure of IVDs detected by T2 mapping and its relation to the physical function showed, in summary, a correlation between the degree of IVDD reflected by lower T2 values in T2 relaxation time in adjacent IVDs and the physical outcome. Patients with lower T2 values showed more physical limitations in everyday life and pain.

Huang et al. [31] compared T2 values of different grades of IVDD in the lumbar spine examined by the Pfirrmann scoring system. They saw significantly lower T2 values in more degenerated IVDs. Müller-Lutz et al. [32] found a high correlation between glycosaminoglycan chemical exchange saturation transfer (gagCEST) imaging and quantitative T2 imaging. They stated that a loss of glycosaminoglycan can lead to low back pain and IVDD. Especially for segments L2/L3 and L3/L4 of the lumbar spine, in the presented group of patients, there were statistically significantly lower T2 values in the inner part of adjacent IVDs compared to nonadjacent ones. This might imply that in those discs with contact to the vertebra after kyphoplasty, the degeneration is accelerated compared to those discs which neighbour vertebral bodies without bone cement augmentation. Like in articular cartilage, the loss of glycosaminoglycan leads to degeneration and pain [33,34]. These findings are underscored by the results presented by Haneder et al. [35]. They described that in sodium imaging, degenerated IVDs proved to have a significant decrease in sodium and hence a loss of glycosaminoglycan.

Using the SF-36, Hoshino et al. [36] examined patients who had undergone balloon kyphoplasty and patients with conservative treatment 6 months after an osteoporotic fracture with poor prognostic factors. They detected an improvement in the subscale VT of SF-36 in patients treated with kyphoplasty compared to conservative treatment [36]. In our study, positive correlations with T2 values of adjacent IVDs were found for the SF-36 items VT, PF, GH and the PCS. We were able to show that with rising T2 values in the IVD, patients seemed to reach higher scores for these items in the SF-36. Thus, higher T2 values might have a positive impact on these items. In our study cohort, especially patients older than 70 years showed fewer limitations for the items VT, GH and the PCS. 

In regard to BMI, Dario et al. [37] showed in their systematic review of twin studies that, statistically, body weight and lumbar IVDD showed a positive correlation in five cross-sectional studies. Several other studies also showed a correlation between IVD herniation and BMI [38,39,40]. Müller-Lutz et al. [32] found a reduction in gagCEST effect and T2 values with increasing BMI. BMI also seemed to have an influence on patients in our study cohort. Here, we found a positive correlation between T2 values of adjacent IVDs and the items VT and GH. It appeared that patients with a BMI less than 25 kg/m^2^ showed higher scores for VT and GH. Thus, patients with normal weight seem to show less degeneration in adjacent IVDs two years after kyphoplasty than overweight patients and, therefore, are more vital and healthier in general.

Using a cadaveric model, the study of Ellingson et al. [41] demonstrated that “T2* mapping is a sensitive method capable of detecting changes associated with disc degeneration. Features of disc health quantified with T2* predicted altered functional mechanics of the lumbar spine better than traditional Pfirrmann grading” [41]. There were numerous previous studies comparing quantitative imaging approaches and the evaluation by Pfirrmann grading which showed a correlation between these methodologies [42]. Therefore, we refrained from using the Pfirrmann grading score. 

Regarding the ODQ, Middendorp et al. [43] examined the association between lumbar IVDD detected by the Pfirrmann grading and the ODI, which can be considered as a summary of the ODQ. Here, they found that with increasing lumbar IVDD, there was an increased ODI [43]. Our study yields similar findings. Patients with a higher score in pain intensity, which means more pain, showed lower values in T2 relaxation time. Hence, patients seem to suffer more pain if they show a higher grade of degeneration in adjacent IVDs. Additionally, regarding personal care, lifting, sitting and standing, we found statistically significant negative correlations with T2 values of adjacent IVDs. This might lead to the interpretation that patients presenting lower T2 values might have problems in personal care and are less able to lift, sit and stand. It may be assumed that the higher the grade of degeneration represented by lower T2 values in adjacent IVDs, the more the clinical outcome and patient’s self-reliance and life seem to be affected. In our study cohort, we saw low mean values in the subscales of the ODQ for the items pain intensity, personal care, lifting, sitting and standing. This might lead to the conclusion that patients’ physical outcome and quality of life two years after kyphoplasty seem to be good. In the EVOLVE Trial by Beall et al. [44], similar findings relating to patients’ life after kyphoplasty were observed. The authors found a reduction in pain and disability and an improvement in physical functioning, personal care and quality of life [44]. Similarly, in another clinical trial, Dohm et al. [45] showed a statistically significant and clinically relevant amendment in pain, disability and quality of life after kyphoplasty.

As for the NRS, in our study cohort, older female patients showed higher scores than the younger ones. This might be explained by increased comorbidity or a higher degree of IVDD in the elderly, which might lead to more pain. For male participants, this correlation could not be shown. Jones et al. [46] found quite similar results regarding low back pain in the elderly. As low back pain is one of the most common geneses for pain in elderly patients, they assumed that this might be caused by reduced activity in daily life [46]. Furthermore, they stated that “the most common physical factors for the development of lower back pain are older age, female gender, obesity, and smoking” [46].

Kaiser et al. [47] conducted research on the correspondence between bone mineral density and IVDD across age and sex. They claimed that there is a difference in bone density in male and female patients with increasing age [47]. In comparison to men, women showed a decrease in bone density twice as quickly not only in the central part of vertebrae, but also in the peripheral zone [47]. This difference in bone density between the inner and outer parts might cause changes in pressure to IVDs and, thus, might result in IVDD. In our study cohort, we were able to find a statistically significant negative correlation between the NRS and T2 values of adjacent IVDs for male patients. This might support the thesis that with increasing degeneration of the adjacent IVDs, patients experience more pain. There might be gender-specific differences in physical outcome, especially regarding pain, and in the predominant grade of IVDD. De Schepper et al. [48] examined the association between lumbar disc degeneration and low back pain in view of the influence of age, gender and individual radiographic features (osteophytes vs. disc space narrowing). They found more osteophytes in men and more disc narrowing in women. The latter seemed to cause more low back pain. There are several other studies examining differences in IVD between females and males [49,50]. In our study, the distribution between women and men was unequal (14 women, 6 men), which is due to the fact that we included consecutive patients in the study and that osteoporotic fractures are more common in female patients. Furthermore, the study cohort was too small for a valid comparison between males and females. Future studies should consider sex-specific differences, as these may have an effect on disc changes.

Despite the interesting results, some limitations of our study should be taken into account. The most ostensible limitation is the number of patients included. Nonetheless, by evaluating a high number of ROIs per patient, the total number of ROIs provided sufficient data for assessment. Conclusions should be drawn cautiously, but the results of this feasibility study may provide a basis for further studies. In addition, there might be a potential selection bias considering that only patients who were mentally and physically able to be examined by MRI two years after kyphoplasty agreed to join the study. Patients who suffered from severe pain or could not remain in a supine position during MRI could not take part in the study. On top of that, patients with high-grade disc bulging were excluded from the start because there would have been a misaligned proportion of the ROIs in favour of ROI 6. However, it was possible to identify patients with different stages of physical fitness and MR morphological grades of IVDD. A further limitation lies in the lack of baseline survey and baseline imaging, which makes it hard to compare the patients’ lives and degree of IVDD before and after kyphoplasty. Because patients came to our care only after they had sustained the fracture, it was not possible to obtain information on whether degeneration was already present before the fracture. Apart from that, there was no possibility to find a control group of patients without any IVDD because patients in this study were mainly advanced in years. Therefore, we had to create an internal control group between adjacent and nonadjacent IVDs in the thoracolumbar spine. Ideally, future studies should be designed as prospective with a control group in which patients did not undergo surgery. 

Moreover, future studies should make sure that, in addition to working with larger study groups, they consider males and females in a similar proportion, because a predominantly female study group might distort the results. Finally, another limitation might be the slice thickness we used for MRI examination. This might have caused the wide standard deviation, e.g., in some of the ROI analysis of T2 relaxation time values. Future studies will target a smaller slice thickness for higher resolution and may consider using ultrashort TE sequencing to capture more precise relaxometry closer to the AF.

## 5. Conclusions

This follow-up feasibility study investigates the clinical physical outcome of patients two years after kyphoplasty regarding IVDD detected by lower T2 relaxation times in quantitative MRI. With this technique, the differentiation between healthy and degenerated IVDs might be improved in future, which will help to develop a better insight into the consequences for the patients’ quality of life and self-reliance after vertebral fractures.

## Figures and Tables

**Table 1:** Inner part (IP) of the intervertebral disc. Mean T2 relaxation time values ± standard deviation (SD) for adjacent and nonadjacent IVDs of the inner part (IP) represented by ROI 2, ROI 3, ROI 4 and ROI 5.

	Segment	Adjacent Vertebral Disc (ms)	Nonadjacent Vertebral Disc (ms)	*p*-Value Difference
ROI 2	TH11/TH12 TH12/L1 L1/L2 L2/L3 L3/L4 L4/L5 L5/S1	74.59 ± 44.99 52.01 ± 15.10 51.66 ± 12.54 48.05 ± 12.86 54.02 ± 16.73 53.01 ± 12.75 56.38 ± 14.89	62.78 ± 21.20 42.38 ± 7.75 60.15 ± 40.61 63.42 ± 42.67 57.71 ± 15.95 54.82 ± 12.00 56.19 ± 15.08	0.920 0.001 0.662 0.001 0.251 0.465 0.991
ROI 3	TH11/TH12 TH12/L1 L1/L2 L2/L3 L3/L4 L4/L5 L5/S1	92.81 ± 70.18 62.56 ± 26.75 58.48 ± 14.11 53.12 ± 12.88 56.60 ± 10.70 55.76 ± 9.12 65.83 ± 31.12	66.10 ± 19.49 55.77 ± 11.46 65.72 ± 31.99 71.85 ± 32.78 65.74 ± 16.39 62.14 ± 14.75 71.44 ± 27.13	0.533 0.514 0.610 0.001 0.031 0.139 0.262
ROI 4	TH11/TH12 TH12/L1 L1/L2 L2/L3 L3/L4 L4/L5 L5/S1	101.87 ± 64.60 64.41 ± 21.02 58.41 ± 12.19 55.99 ± 12.23 53.84 ± 10.75 55.20 ± 7.29 56.46 ± 20.06	68.63 ± 19.62 58.59 ± 14.01 63.71 ± 19.48 74.21 ± 35.20 68.12 ± 22.92 63.67 ± 15.41 68.25 ± 22.32	0.262 0.391 0.456 0.002 0.008 0.069 0.052
ROI 5	TH11/TH12 TH12/L1 L1/L2 L2/L3 L3/L4 L4/L5 L5/S1	108.07 ± 97.42 59.69 ± 18.95 50.54 ± 11.65 48.28 ± 9.51 49.50 ± 9.55 54.58 ± 4.65 55.28 ± 16.15	69.88 ± 14.93 54.83 ± 10.91 55.10 ± 13.05 59.61 ± 12.78 57.04 ± 11.68 57.56 ± 10.43 65.86 ± 17.65	0.516 0.514 0.169 0.000 0.007 0.388 0.075

ROI 2 and ROI 5 represent the intermediate zone (IZ) between the anulus fibrosus (AF) and the nucleus pulposus (NP), which is represented by ROI 3 and ROI 4. Each ROI is subcategorised by the segments from TH11/Th12 to L5/S1. *p*-values and SD are given for the difference between adjacent and nonadjacent IVDs (*p*-values ≤ 0.05 are labelled in boldface).

**Table 2:** Outer part (OP) of the intervertebral disc. Mean T2 relaxation time values ± standard deviation (SD) for adjacent and nonadjacent IVDs of the outer part (OP) represented by ROI 1 and ROI 6.

	Segment	Adjacent Vertebral Disc (ms)	Nonadjacent Vertebral Disc (ms)	*p*-Value Difference
ROI 1	TH11/TH12 TH12/L1 L1/L2 L2/L3 L3/L4 L4/L5 L5/S1	80.75 ± 48.62 59.99 ± 45.25 51.27 ± 13.51 53.81 ± 14.73 56.33 ± 29.40 53.65 ± 14.78 49.36 ± 8.40	67.04 ± 22.11 45.33 ± 15.43 55.43 ± 24.11 62.89 ± 37.41 68.19 ± 47.84 52.74 ± 18.70 51.63 ± 19.31	0.867 0.001 0.882 0.646 0.063 0.516 0.829
ROI 6	TH11/TH12 TH12/L1 L1/L2 L2/L3 L3/L4 L4/L5 L5/S1	109.69 ± 113.61 69.80 ± 42.13 58.04 ± 21.98 48.50 ± 12.32 49.31 ± 12.39 60.05 ± 17.75 64.31 ± 21.69	73.32 ± 27.57 70.34 ± 75.77 54.87 ± 25.69 56.93 ± 30.46 60.18 ± 39.46 54.68 ± 17.43 70.75 ± 56.65	0.899 0.044 0.416 0.542 0.324 0.232 0.866

Each ROI is subcategorised by the segments from TH11/Th12 to L5/S1. *p*-values and SD are given for the difference between adjacent and nonadjacent IVDs (*p*-values ≤ 0.05 are labelled in boldface).

**Table 3:** Mean values of 36-Item Short Form Survey.

SF-36 Subscale	Minimum	Maximum	Mean Value	SD
VT	15	100	56.25	24.60
PF	20	100	50.00	27.74
BP	33	100	59.25	23.41
GH	30	95	56.75	17.72
RP	0	100	50.00	46.60
RE	0	100	78.33	36.31
SF	13	100	80.62	27.35
MH	48	100	75.20	15.82
PCS	33	70	50.00	10.77
MCS	29	61	50.00	8.97

Minimum, maximum and mean values ± standard deviation (SD) of the subscales of the SF-36. VT (vitality), PF (physical functioning), BP (bodily pain), GH (general health perception), RP (physical role functioning), RE (emotional role functioning), SF (social role functioning), MH (mental health), PCS (physical component summary), MCS (mental component summary).

**Table 4:** Mean values of the Oswestry Low Back Pain Disability Questionnaire (ODQ) and Disability Score (ODI).

ODQ Subscale	n	Minimum	Maximum	Mean Value	SD
Pain intensity	20	0	3	1.05	1.15
Personal care	20	0	2	0.60	0.598
Lifting	20	0	4	1.75	1.55
Walking	20	0	5	1.30	1.49
Sitting	20	0	5	1.10	1.37
Standing	20	0	4	1.85	1.57
Sleeping	20	0	3	0.40	0.75
Sex life	12	0	5	0.75	1.60
Social life	20	0	4	1.00	1.34
Travelling	20	0	5	1.45	1.79
ODI	20	0	73.33	28.09	22.80

Minimum, maximum and mean values of the items of the ODQ ± standard deviation (SD). n describes the number of patients who answered the question belonging to the subscale of the ODQ. The ODI is indicated in %.
